# Structure of Starch–Sepiolite Bio-Nanocomposites: Effect of Processing and Matrix–Filler Interactions

**DOI:** 10.3390/polym15051207

**Published:** 2023-02-27

**Authors:** Daniele Bugnotti, Sara Dalle Vacche, Leandro Hernan Esposito, Emanuela Callone, Sara Fernanda Orsini, Riccardo Ceccato, Massimiliano D’Arienzo, Roberta Bongiovanni, Sandra Dirè, Alessandra Vitale

**Affiliations:** 1 Department of Industrial Engineering, University of Trento, 38123 Trento, Italy; 2 Department of Applied Science and Technology, Politecnico di Torino, 10129 Torino, Italy; 3 INSTM-Politecnico di Torino Research Unit, 50121 Firenze, Italy; 4 Department of Materials Science, University of Milano-Bicocca, 20125 Milano, Italy

**Keywords:** yuca starch, plasticized starch, sepiolite filler, crystalline structure, bio-composite, nanocomposite

## Abstract

Sepiolite clay is a natural filler particularly suitable to be used with polysaccharide matrices (e.g., in starch-based bio-nanocomposites), increasing their attractiveness for a wide range of applications, such as packaging. Herein, the effect of the processing (i.e., starch gelatinization, addition of glycerol as plasticizer, casting to obtain films) and of the sepiolite filler amount on the microstructure of starch-based nanocomposites was investigated by SS-NMR (solid-state nuclear magnetic resonance), XRD (X-ray diffraction) and FTIR (Fourier-transform infrared) spectroscopy. Morphology, transparency and thermal stability were then assessed by SEM (scanning electron microscope), TGA (thermogravimetric analysis) and UV–visible spectroscopy. It was demonstrated that the processing method allowed to disrupt the rigid lattice structure of semicrystalline starch and thus obtain amorphous flexible films, with high transparency and good thermal resistance. Moreover, the microstructure of the bio-nanocomposites was found to intrinsically depend on complex interactions among sepiolite, glycerol and starch chains, which are also supposed to affect the final properties of the starch–sepiolite composite materials.

## 1. Introduction

Starch is a biopolymer composed of two D-glucose homopolymers: amylose, characterized by linear chains based on α-D(1-4)-glucan bonds, and amylopectin, which is essentially a highly branched amylose through α-D(1-6) links [1]. Depending on the natural source, the amylopectin content is about 60–90% [1]. In the case of yuca starch, the amount of amylose is about 20% [2]. Starch is found in any plant stored in grains, tubers and roots in the form of semicrystalline granules which possess a hierarchical structure made of concentric rings, representing the alternation of crystalline and amorphous regions. The amorphous region is a mixture of amylose, amylopectin branching points and amylopectin, whereas the crystalline region is mainly formed by amylopectin chains and characterized by different polymorphs [1]. Starch represents an ecological alternative to common petroleum-based plastics, due to its high availability, low cost, good biocompatibility and high biodegradability; moreover, having a moderate oxygen permeability, it may be a promising material for packaging [3,4]. However, due to its crystallinity, film-forming ability, transparency and flexibility are not assured. Moreover, films can show relatively poor mechanical properties while the high hydrophilicity causes low stability in aqueous media and low water barrier properties.

**Figure 1:**
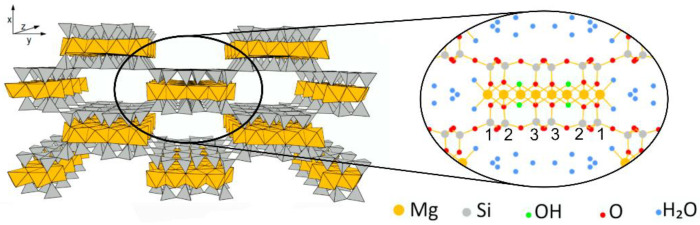
Sepiolite structure. Numbers refer to silicon atom positions (Si1, Si2, Si3). Figure adapted from [21].

Thus, as a mitigation strategy of the limited performance of starch films in terms of mechanical and barrier properties, composites can be prepared. An interesting natural filler is sepiolite [5], a magnesium silicate clay of particular interest due to its low cost and high availability, remarkable chemical and mechanical stability and anisotropic particle shape [6]. The structure of sepiolite is reported to be an octahedral Mg(II) sheet embedded by two layers of tetrahedral SiO_4_, giving the chemical formula [Si_12_O_30_Mg_8_ (OH)_4_(OH_2_)_4_ nH_2_O]. In sepiolite, the edges are shared with the neighboring ones, resulting in a “checkerboard” pattern with the remarkable formation of tunnels (Figure 1). Inside these tunnels, two water molecules (structural water) are coordinated to each of the external Mg ions and hydrogen bonded to zeolitic water molecules. This particular configuration provides excellent adsorptive properties to sepiolite clay [7]. For instance, as concerns on food packaging applications, a high ethylene adsorptive efficiency was reported [8]. Ethylene is a plant hormone that causes quick ripening and easily alters fresh products, along with microbial growth; thus, in packaging technology, sepiolite could act as an ethylene scavenger/adsorber replacing either unsafe oxidizing materials (e.g., metals, potassium permanganate) or non-biobased nanotubes and carbon dots [9,10], suggesting that sepiolite composite films could be exploited for active packaging.

Sepiolite can be combined with starch to form bio-nanocomposites by different techniques, such as solvent exchange process [11], dry-blend process with organo-modified sepiolite [12] or mechanical mixing and ultrasonication in water [13]. Sepiolite nanofillers have been demonstrated to have a good reinforcing effect, to improve water resistance and to reduce the water absorption of starch, allowing attractive bio-composites to be obtained for a wide range of applications [14,15,16]. In particular, as the silanol groups of the sepiolite clay can form hydrogen bonds with the hydroxyl groups of starch [12], the interactions at the filler–matrix interface are expected to greatly influence the final properties of the nanocomposites [17,18,19,20]. However, such interactions in starch–sepiolite bio-nanocomposites have not yet been deeply studied in the literature.

In this research work, composite films made of starch as the matrix and sepiolite as the filler were produced and characterized, particularly studying the effect of the processing and of the introduction of sepiolite filler on the structure of the starch-based films, to lay the background for the development of sustainable materials potentially suitable for packaging applications. Starch gelatinization and the addition of glycerol were used to obtain flexible films by disrupting the rigid lattice structure of semicrystalline starch. Mechanical mixing in water was chosen as a processing route to obtain a homogeneous dispersion of sepiolite in starch solutions. The structure and morphology of the polymer were investigated by SS-NMR (solid-state nuclear magnetic resonance), XRD (X-ray diffraction), FTIR (Fourier-transform infrared) spectroscopy, and SEM (scanning electron microscope). Thermal stability and transparency of the composite films were investigated by TGA (thermogravimetric analysis) and UV–Vis spectroscopy, respectively.

## 2. Materials and Methods

### 2.1. Materials

A yuca starch powder originated from Colombia was used. Glycerol (≥99.0%) was purchased from Sigma-Aldrich (Milan, Italy).

Sepiolite clay Pangel S9 (40–150 nm width and 1–10 μm length, Figure S1 in the Supplementary Material) was provided by Tolsa (Madrid, Spain) and extracted from the landfill of Vallecas (Madrid, Spain).

### 2.2. Samples Formulations and Preparation

First, 2.5% *w*/*w* starch solution in water was prepared by stirring at 70 °C for 30 min to achieve gelatinization. The solution was cast onto a polystyrene Petri dish and oven-dried at 40 °C. The film obtained was labeled Y_f_. As a reference, yuca starch in form of powder (Y_p_) was also analyzed.

Plasticized starch films (YG_f_) were prepared by adding 40% *w*/*w* of glycerol with respect to starch before proceeding with gelatinization as described above.

To prepare nanocomposite film samples with different filler contents (i.e., 3, 5, 10 and 15% *w*/*w* with respect to starch), sepiolite was suspended in water and mixed overnight with a magnetic stirrer at room temperature prior to starch and glycerol addition. The suspension was then stirred at 70 °C for 30 min to achieve gelatinization. The film-forming solution obtained in this way was cast onto a polystyrene Petri dish and oven-dried at 40 °C. The obtained films were about 50 µm thick. Samples labels and compositions are reported in Table 1.

In addition, two film samples of starch and sepiolite (at 5% and 15% *w*/*w* with respect to starch, namely Y5S_f_ and Y15S_f_) were manufactured without glycerol and were analyzed only by ^29^Si CPMAS NMR.

Finally, an amorphous standard sample for crystallinity determination was prepared by boiling a 0.01 g/mL starch suspension in water for 30 min and oven-dried at 60 °C overnight.

### 2.3. Structural Characterization

#### 2.3.1. Solid-State Nuclear Magnetic Resonance

SS-NMR spectra of the samples were recorded with a Bruker (Billerica, MA, USA) 400WB spectrometer operating at a proton frequency of 400.13 MHz under the following conditions for cross polarization magic angle spinning (CPMAS) experiments: ^13^C frequency: 100.48 MHz, contact time 1.5 ms, decoupling length 5.6 µs, recycle delay 3 s, 2 k scans. ^29^Si frequency: 79.48 MHz, contact time 5 ms, decoupling length 6 μs, recycle delay 8 s, 10 k scans. Samples were packed in zirconia rotors, which were spun at 7 kHz. Adamantane and Q_8_M_8_ were used as external secondary references. Crystallinity was evaluated following the methodology of Tan et al. [22]. Line shape analyses of NMR spectra were carried out with Bruker TopSpin software (version 3.6.4). The results were considered acceptable with confidence level > 95%.

#### 2.3.2. X-ray Diffraction

Long-range order was analyzed by means of XRD analyses performed on a Rigaku (Tokyo, Japan) DMAX III diffractometer in Bragg–Brentano geometry, equipped with a Cu source (λ = 1.54056 Å) in the following conditions: 2θ range from 2° to 45°, steps of 0.05° and 3 s counting time. Crystallinity was evaluated following the methodology proposed by Lopez-Rubio et al. [23]. In order to precisely measure *d*-spacing in sepiolite composites, XRD was also carried out in asymmetric scattering with a fixed incident angle of 1.5°, with the other parameters unchanged. In this case the film was fixed to the sample holder by a small piece of modeling clay to avoid misalignment. The asymmetric scattering was necessary to eliminate any reflections that would have come from the modeling clay.

Profile fitting analysis of XRD patterns were performed with Origin Pro 2018. Results were considered acceptable with R^2^ > 0.98.

#### 2.3.3. Fourier-Transform Infrared and UV–Vis Spectroscopy

Changes in conformational order were investigated by Fourier-transform infrared spectroscopy (FTIR) in attenuated total reflectance (ATR) mode on a Nicolet iS50 spectrometer (Thermo Fisher Scientific Inc., Waltham, MA, USA), equipped with a Smart iTX-Diamond ATR accessory. Spectra were averaged over 32 scans in the 4000–550 cm^−1^ range, with 4 cm^−1^ resolution. The crystalline-sensitive region of the spectra 1200–800 cm^−1^ was isolated, baseline corrected with a straight line and fitted with Gaussian peaks; then, the area ratio of 1040 cm^−1^/1016 cm^−1^ peaks was calculated.

Profile fitting analysis of FTIR-ATR spectra was performed with Origin Pro 2018. Results were considered acceptable with R^2^ > 0.98.

UV–Vis spectroscopy was carried out with a JENWAY 6850 UV/Vis spectrophotometer (Cole-Parmer, Stone, Staffordshire, UK).

#### 2.3.4. Scanning Electron Microscopy

SEM analyses were performed by a Vega TS5136 XM Tescan (Milan, Italy) microscope in a high-vacuum configuration. The electron beam excitation was 30 kV at a beam current of 25 pA, and the working distance was 12 mm. In this configuration, the beam spot was 38 nm. The samples were applied on carbon tape onto an aluminum substrate and covered with gold coating.

#### 2.3.5. Thermogravimetric Analysis

The TGA was performed using a Mettler Toledo (Milan, Italy) TGA/DSC1 STARe system under N_2_ flux. The materials were equilibrated at 30 °C for 15 min and then heated from 30 to 1000 °C at the rate of 10 °C min^−1^.

## 3. Results and Discussion

### 3.1. Effect of Processing on the Crystalline Structure of Starch

The film-forming solution was prepared by gelatinization, a process in which the suspension of starch granules in water undergoes the action of temperature and the swelling of amorphous regions, resulting in destruction of crystalline domains [24]. Through SS-NMR, XRD and FTIR-ATR analyses, the amorphization and plasticization of yuca starch was investigated.

**Figure 2:**
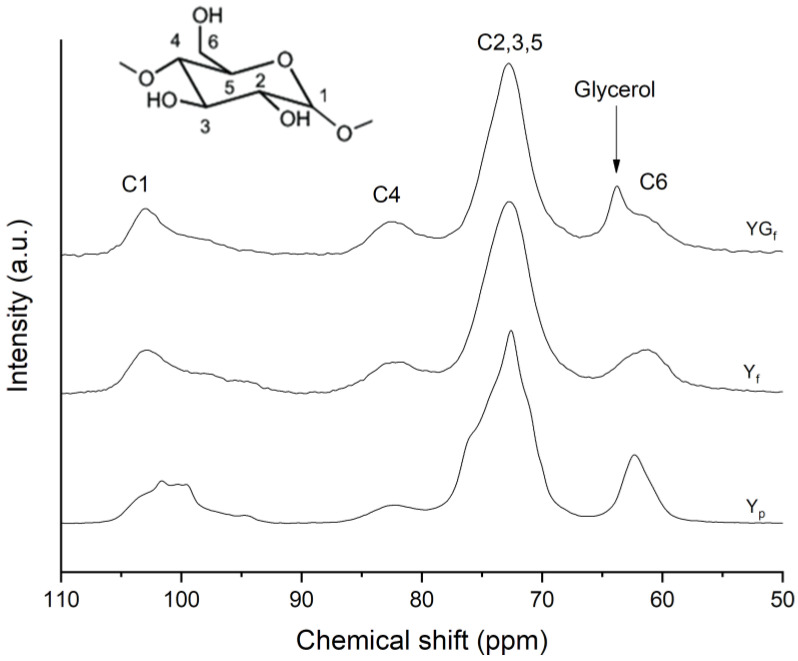
^13^C CPMAS NMR spectra of Y_p_, Y_f_ and YG_f_ samples. The arrow indicates the glycerol methylene peak.

The ^13^C CPMAS NMR spectra of pristine starch powder (Y_p_), starch film (Y_f_) and plasticized starch film (YG_f_) samples are reported in Figure 2, with carbon labels specified in the inset. C1 carbon resonance is detected at 94–105 ppm, C4 at about 80–84 ppm, C2,3,5 resonances give rise to a complex band in the range 68–77 ppm and C6 in the 58–64 ppm region, as also reported in the literature [25].

The most interesting sites are C1 and C4, as they are involved in glycosidic bonds, and they are the most sensitive to starch conformations, since they are defined by the geometry of glycosidic linkages [25,26]. As a matter of fact, the C1 region showed differences between Y_p_ and Y_f_ and YG_f_ film samples. Y_p_ was characterized by a shoulder at 102.9 ppm attributed to a V-type polymorph [22,26] and the typical triplet signal of an A-type polymorph at 101.6, 100.3 and 99.6 ppm [22,25]. From the profile-fitting analysis of the C1 signal (Figure S2 in the Supplementary Material), double helices accounted for 44.4% and V-type single helix component for 2.5%. Y_f_ and YG_f_ film presented a completely different shape of C1 site, typical of amorphous starches [23,26].

The line shape of C2,3,5 substantially changed as the components merged together into a broader and less resolved signal. It is worth noting that the C4 site, the intensity of which relates to the amorphous part of starch [25], increased in intensity. These features indicated the amorphization of starch as a consequence of film processing. In addition, the YG_f_ sample presented glycerol resonances, namely CH_2_-OH at 63.8 ppm and the CH-OH at 72 ppm, which is overlapped to C2,3,5 signals of starch [27,28].

**Figure 3:**
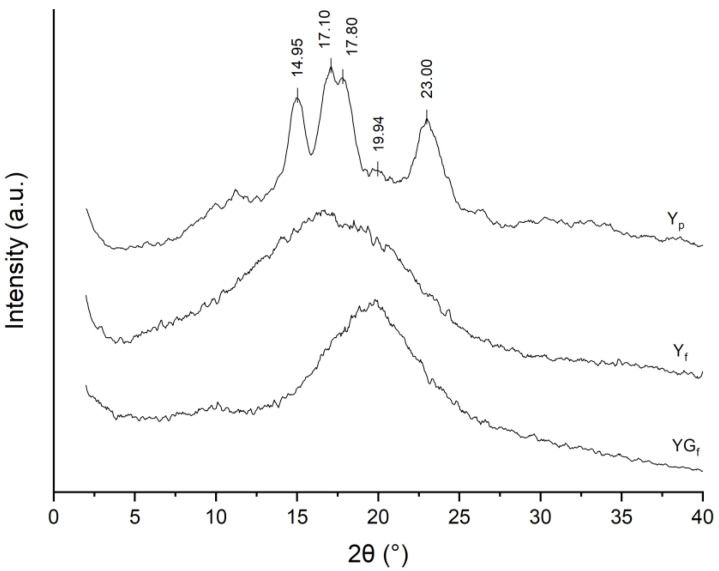
XRD patterns of Y_p_, Y_f_ and YG_f_ samples.

From XRD patterns (Figure 3), the typical reflections of the A-type polymorph were found in Y_p_ at 2θ 9.97°, 11.24°, 14.95°, 17.10°, 17.80°, 23.00° and 26.42°, with the V-type reflection at 2θ 19.94° [23]. Profile-fitting analysis of the Y_p_ pattern (Figure S3 in the Supplementary Material) resulted in a crystallinity equal to 45.2%, while the V-type content was 1.5%. These results are in good agreement with the NMR analysis. Moreover, the higher V-type content determined through NMR highlighted the presence of single helices in the amorphous domain [23].

XRD patterns of Y_f_ and YG_f_ samples (Figure 3) were characterized by an amorphous halo, with the loss of crystalline features due to amorphization, confirming NMR results. The different position and intensity of the amorphous halo in YG_f_ could be explained by chain–glycerol interactions.

**Figure 4:**
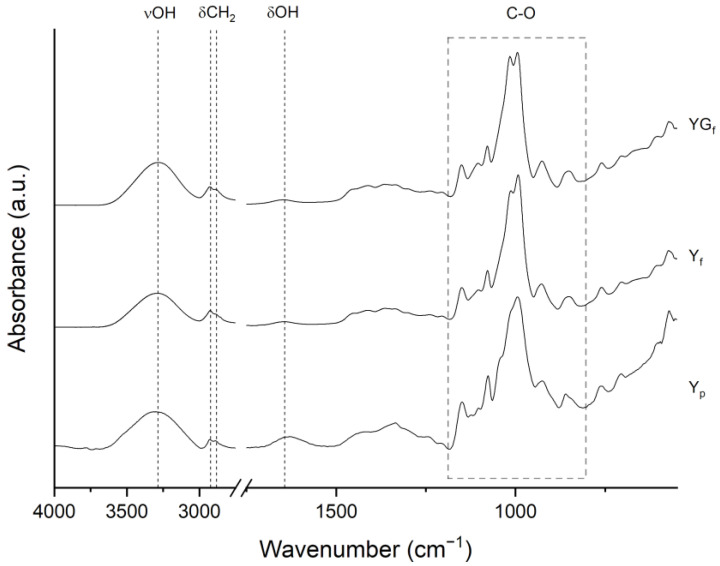
FTIR-ATR spectra of Y_p_, Y_f_ and YG_f_ samples. Main bond vibrations are indicated.

Comparing the FTIR-ATR spectra of Y_p_ to those of film samples (Figure 4), the effect of processing is also evident. In Figure 4 the spectra are reported, and main peaks assignments are listed in Table S1 in the Supplementary Material. The broad band centered at 3307 cm^−1^ and the signal at about 1630 cm^−1^ are assigned to O-H stretching vibrations and water scissoring, respectively. In film samples prepared with and without glycerol, the O-H stretching band shifted to 3282 cm^−1^, indicating strong hydrogen bonding between plasticizer molecules (water and glycerol) and starch macromolecules [29]. The CH_2_ stretching vibrations were clearly visible with peaks at 2925 (C-H asymmetric stretching) and 2884 cm^−1^ (C-H symmetric stretching). In the 1500–1200 cm^−1^ range, the overlapped bands of CH_2_ bending and some COH vibrations are found. The most interesting region is the one delimited by the dashed rectangle in Figure 4 (1200–800 cm^−1^), characteristic of the C-O vibrations in COH and COC groups of starch. Particularly, peaks at 1150, 1125 (barely visible in processed starches) and 1103 cm^−1^ are related to C-O, C-C and C-OH stretching modes, while signals at 1076, 1040, 1016, 992 and 926 cm^−1^ are due to C-O-H bending and CH_2_-related modes [30,31,32]. Precise assignment of every peak in this region is not possible due to poorly resolved and overlapped bands [30,31]. However, this spectral region is of particular importance as it is sensitive to starch crystalline and amorphous conformations [33]. In detail, the peaks at 992 and 1040 cm^−1^ are related to crystalline domains, while the peak at 1016 cm^−1^ is related to the amorphous regions [30,31,33].

In Y_p_, a shoulder was clearly visible at 1045 cm^−1^, while for film samples it was much less pronounced, with a shift toward 1040 cm^−1^. Moreover, for film samples, the 1016 cm^−1^ peak increased in intensity with respect to the 992 cm^−1^ peak. These differences suggested structural modifications as a consequence of processing.

An FTIR profile-fitting analysis was carried out to better highlight the peaks attributed to crystalline and amorphous structures (as an example, the FTIR profile fitting for Y_p_ is reported in Figure S4 of the Supplementary Material). Ratios of intensities or integrated areas of the characteristic peaks (e.g., 1040/1016, 992/1016) are usually exploited as an indicator of the degree of ordered structures in starch samples. However, correlations between XRD and FTIR measurements of long-range order are very weak [30,34], and the analysis is hindered by the hydration sensitivity of 1016 and 992 cm^−1^ peaks, which can alter their intensity ratio [31,32]. In this study, the analysis was carried out only to highlight differences between Y_p_ and film samples with or without fillers. Results of 1040/1016 peak ratio (Figure S5 in the Supplementary Material) indicated different values between Y_p_ (1.77), Y_f_ (0.80) and YG_f_ film (1.01), in line with starch amorphization, as a consequence of film processing.

In conclusion, NMR, XRD and FTIR analyses highlight changes in molecular conformations after starch processing.

### 3.2. Effect of Sepiolite Addition on Film Microstructure

**Figure 5:**
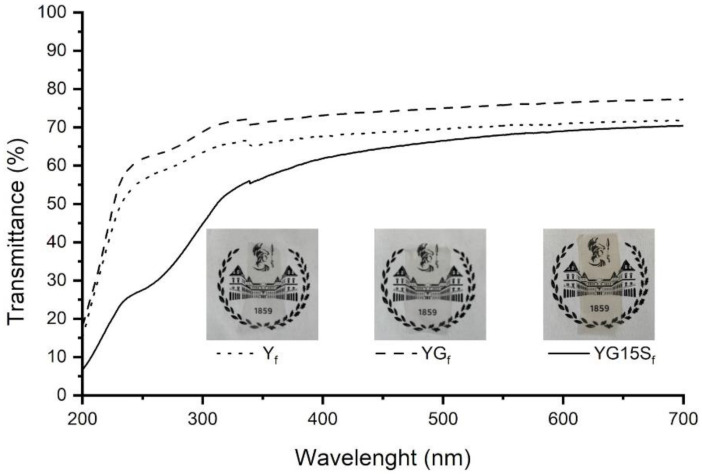
UV–Vis transmittance spectra and pictures of starch film (Y_f_), plasticized starch film (YG_f_) and nanocomposite films with 15% *w*/*w* of sepiolite (YG15S_f_).

Starch-based nanocomposite films were prepared by adding sepiolite in different contents (i.e., 3, 5, 10 and 15% *w*/*w* with respect to starch). Uniform and transparent films were obtained (Figure 5), independently on the filler amount. The presence of sepiolite imparted a yellowish color to the films, which was more intense in the case of a higher filler content. The transparency of the samples was analyzed by UV–Vis spectroscopy: the acquired spectra are shown in Figure 5. To assess the effect of sepiolite, the film with the highest amount of filler (YG15S_f_) was compared with a starch film (Y_f_) and a plasticized starch film (YG_f_). In the visible range, the effect of the addition of glycerol or sepiolite is negligible. In YG15S_f_, an absorbance peak below 300 nm is detected, due to sepiolite Mg-O-Si bonds [35].

**Figure 6:**
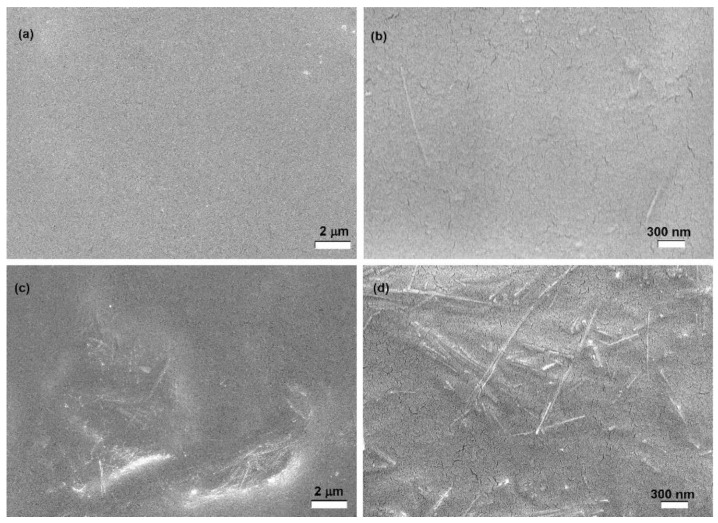
SEM images of YG3S_f_ (**a**,**b**) (10 kX and 50 kX magnification, respectively) and of YG15S_f_ (**c**,**d**) (10 kX and 50 kX magnification, respectively).

The morphology of the composite films was analyzed by SEM. As an example, Figure 6 reports SEM images of YG3S_f_ and YG15S_f_ films (i.e., lowest and highest value of filler content). The sepiolite clay filler, with its characteristic needle-like shape, is well-dispersed in the starch matrix, and its original dimensions are retained in the composite films. However, some agglomerates could be detected when 15% of sepiolite was introduced (Figure 6c,d).

**Figure 7:**
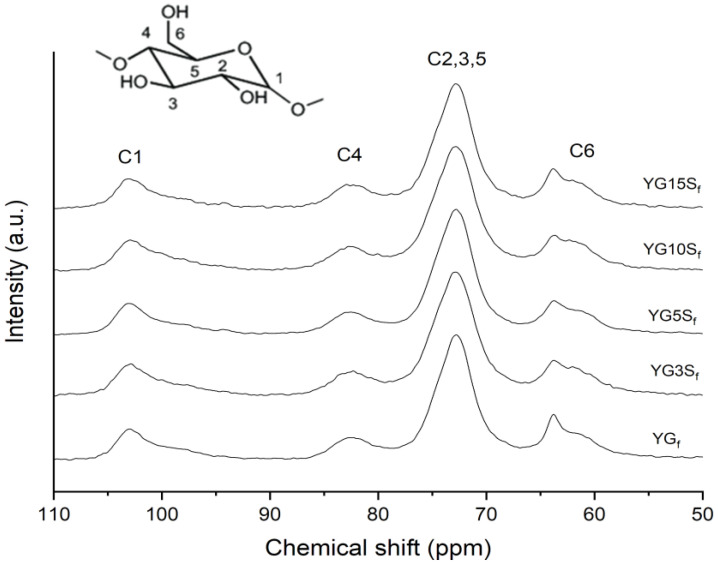
^13^C CPMAS NMR spectra of plasticized starch film (YG_f_) and of composite films with sepiolite at different loadings (YG3S_f_, YG5S_f_, YG10S_f_ and YG15S_f_).

The microstructure of the nanocomposite films was investigated by SS NMR, XRD and FTIR-ATR analyses. The ^13^C CPMAS NMR spectra of composites loaded with sepiolite, together with that of YG_f_ as a reference, are reported in Figure 7. Line shapes of carbon peaks C1, C4 and C2,3,5 were almost identical to those of YG_f_, suggesting that sepiolite did not affect the starch matrix structural conformation, which remained amorphous. A profile-fitting analysis of the C6 site was carried out for each sample. It was observed that glycerol CH_2_-OH resonance position remained constant (centered at 63.9 ppm), but the peak became broader in the composite films with a reduction in intensity compared to the YG_f_ sample. The proportion between the C6 area and glycerol peak remained almost constant among the composite samples; however, the broadening of the glycerol CH_2_-OH peak might indicate less chain mobility or increased anisotropy compared to YG_f_ film, suggesting glycerol–sepiolite interactions [27].

**Figure 8:**
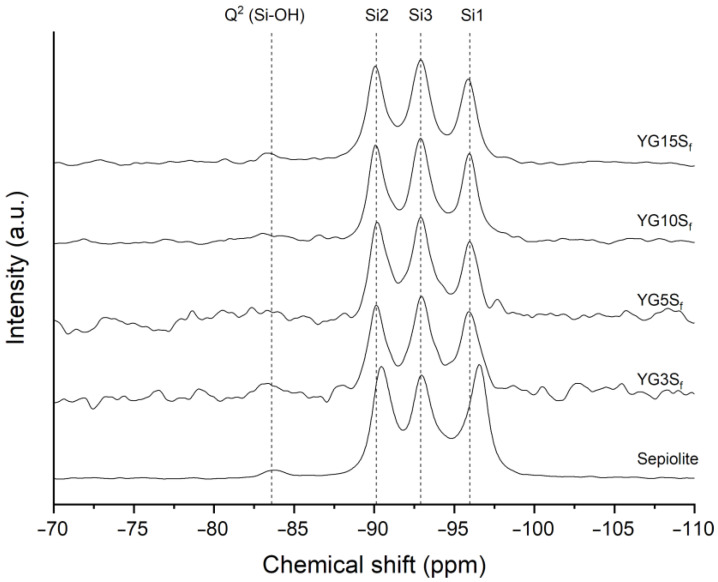
^29^Si CPMAS NMR spectra of pristine sepiolite in comparison with nanocomposite films with sepiolite at different loadings (YG3S_f_, YG5S_f_, YG10S_f_ and YG15S_f_).

Silicon CPMAS NMR was also employed to analyze sepiolite in nanocomposite films. The spectra are reported in Figure 8, together with that of neat sepiolite for comparison. Assignments and labelling of silicon atoms were performed according to the literature [6,36], where Q^n^ describes the SiO_4_ unit with n Si-O-Si oxo-bridges. Briefly, sepiolite structure is characterized by four types of silicon atoms: one Q^2^ and three Q^3^. The three well-resolved resonances accounting for Q^3^ Si atoms correspond to their different position (see Figure 1): edge (Si1) at −96.6 ppm, center (Si3) at −92.9 ppm and near edge (Si2) at −90.4 ppm.

In the composite films, the Si1 peak, located at the edges of the octahedral sheets and very close to structural water, decreased in intensity with respect to neat sepiolite and downfield shifted of about 0.6 ppm. While the intensity ratio of the Q^3^ resonances in the sepiolite sample was about 1:1:1, in the composite films the efficiency of magnetization transfer from protons to Si atoms had changed, modifying the intensity ratio of the three peaks. One possible explanation for such effect could be the substitution of zeolitic water inside sepiolite’s channel with glycerol molecules. Glycerol could undergo polarization exchange with structural water or Mg-OH protons, changing the intensity ratio. This explanation was described by Weir and coworkers in the case of substitution of zeolitic water with D_2_O [36]. Zeolitic water can also be substituted with small organic molecules (e.g., acetone) inside sepiolite’s channels, as reported in the literature [5,7]. The ^29^Si CPMAS NMR spectra of the cited works were very similar to the spectra of the nanocomposite films with sepiolite herein investigated. It is worth noting that the removal of zeolitic water by thermal treatment at 120 °C is not mandatory prior to its substitution; small polar molecules such as methanol, ethanol, ammonia or pyridine can enter sepiolite’s tunnel by displacing zeolitic water molecules [7].

Driven by these hypotheses, two film samples of starch and sepiolite (at 5% and 15% *w*/*w* with respect to starch, namely Y5S_f_ and Y15S_f_) were manufactured without glycerol to study the shape of the Si1 peak. The ^29^Si CPMAS NMR spectra of the composites, reported in Figure S6 of the Supplementary Material, were comparable with the one of neat sepiolite. This indicated that starch could not interact with sepiolite’s channel. Therefore, it was confirmed that the presence of glycerol caused the decrement of Si1 intensity, due to glycerol intercalation in sepiolite’s channel as previously hypothesized.

**Figure 9:**
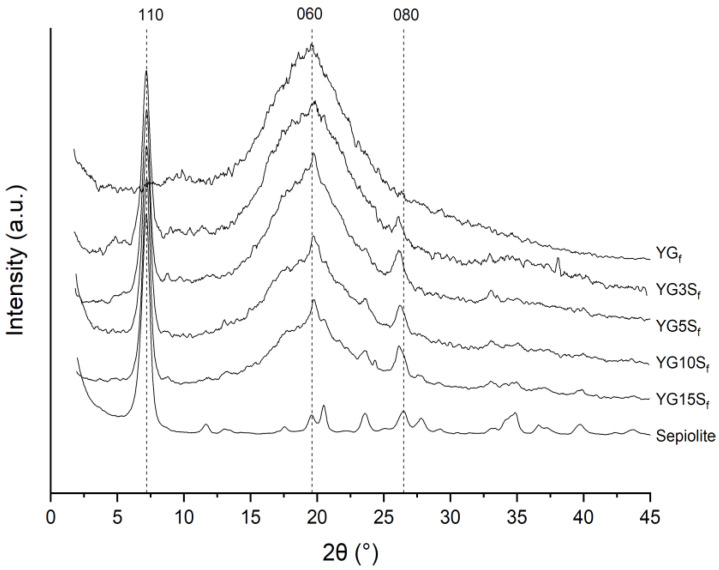
Diffractograms of pristine sepiolite, plasticized starch film (YG_f_) and nanocomposites films with sepiolite at different loadings (YG3S_f_, YG5S_f_, YG10S_f_ and YG15S_f_).

Long-range order of nanocomposite films was investigated by XRD. From the diffractograms reported in Figure 9, the basal plane (110) of sepiolite (PDF card n. 13-595) was centered at about 7.20° (corresponding to a d-space of 1.22 nm from Bragg’s law), and it was clearly visible in all the composite films. Glycerol and starch did not alter the internal channels of sepiolite, which is consistent with the fact that sepiolite’s sheets are kept together by covalent bonds, and it cannot be exfoliated [6]. Other sepiolite reflections were detected at 2θ 19.8°, 20.5° (very weak), 23.6° and 26.3°, which were comparable with the standard (PDF card n. 13-595). In addition, the crystallite size of sepiolite (12 ± 1 nm, from Scherrer’s equation) did not show variations in the nanocomposites.

As expected, the observed peaks increased in intensity with increasing the sepiolite content, but it is worth noting the relative intensity of (060) reflections at 19.8° in the film samples, which appears higher than in pristine sepiolite.

A similar behavior is shown by the (080) peak at 26.3°. It should be mentioned that Chivrac et al. [12] attributed the peak at 26.3° to the formation of a new amylopectin crystalline structure at the filler interface, and they reported this effect likely to be induced by the interaction of silanol groups at the edges of sepiolite needles with hydroxyl groups of starch. A similar effect was described in starch nanocomposites loaded with halloysite clay [29], and in synthetic polymer matrices, sepiolite has been reported to induce preferential orientation in the matrix {0k0} planes or the formation of different polymorphs [12]. However, in our samples no characteristic peaks of crystalline starch were detected, meaning that the amorphous structure of starch did not change upon sepiolite addition, in agreement with NMR results. Analyzing the sepiolite filler and considering the evolution of both (060) and (080) planes with the sepiolite loading, the increased intensity for {0k0} reflections along with the unchanged filler crystallite dimensions and the ^13^C CPMAS NMR results suggest the occurrence of the onset of filler-preferred orientation in the bio-composites. This could be due to interactions with starch leading to sepiolite arrangement into the matrix, as recently reported by some of us for sepiolite–rubber nanocomposites [6,37].

**Figure 10:**
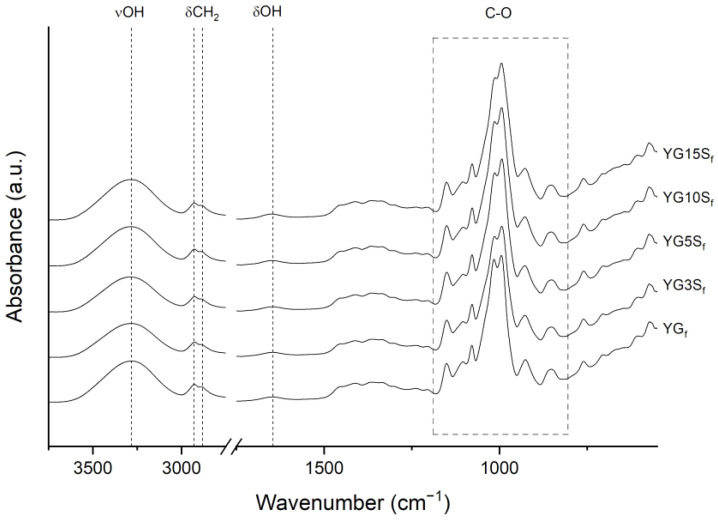
FTIR-ATR spectra of plasticized starch film (YG_f_) and nanocomposite films with sepiolite at different loadings (YG3S_f_, YG5S_f_, YG10S_f_ and YG15S_f_).

From FTIR-ATR analyses, the spectra of the composites resulted very similar to the one of YG_f_ (Figure 10). Profile fitting of the 1200–800 cm^−1^ region further highlighted the similarity of the samples; 1040/1016 area ratios (Figure S5 in the Supplementary Material) were comparable in all the samples, and a clear trend was not observed. From these results, it was concluded that the addition of sepiolite at any investigated amount did not change starch conformation in an appreciable manner, in agreement with NMR and XRD results. As a final remark, sepiolite typical vibrations (MgOH (3700–3555 cm^−1^), Si-O (1057 and 1000 cm^−1^), SiOH (976 cm^−1^) and MgOH (689 cm^−1^) [6], as shown in the spectrum in Figure S7 of the Supplementary Material) could not be identified in the spectra of the bio-composites (Figure 10), due to the overlapping of the signals with starch vibrations. Nevertheless, during profile fitting of the crystalline sensitive region, the integrated area of the peak at about 970 cm^−1^ increased with increasing of sepiolite content (it was three times higher in the YG15S_f_ film than the YG3S_f_ film). This might be explained by the increasing presence of Si-OH bond vibration, possibly confirming that some signals were hidden by starch vibrations.

### 3.3. Thermal Resistance of Nanocomposite Films

**Figure 11:**
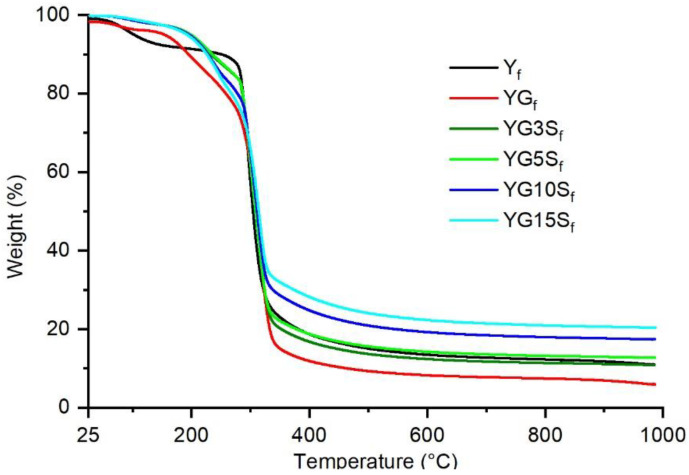
Thermograms of starch film (Y_f_), plasticized starch film (YG_f_) and nanocomposite films with different loadings of sepiolite (YG3S_f_, YG5S_f_, YG10S_f_ and YG15S_f_).

The results of the thermogravimetric analysis performed on the film samples are shown in Figure 11. Table 2 reports the decomposition temperatures T_5_ and T_20_, corresponding to the temperature at which the sample weight losses are 5% and 20%, respectively, and the residual weights.

The non-plasticized unfilled starch film sample, Y_f_, showed two main weight loss events, the first close to 100 °C, due to loss of adsorbed water, and the second around 300 °C, attributed to the decomposition of starch. The glycerol containing films showed a reduced amount of adsorbed water. However, an additional weight loss event, between 150 °C and 260 °C, appeared, lowering the thermal stability of the plasticized films YG_f_ with respect to the non-plasticized starch film Y_f_, as demonstrated by the lower T_20_ (Table 2). Such behavior was consistent with that observed in other studies of plasticized starch films [38] and was attributed to the decomposition of glycerol [39,40]. The composite films containing sepiolite showed a better thermal resistance compared to YG_f_, increasing the T_5_ of around 45 °C. However, by increasing the amount of sepiolite, the thermal stability slightly decreases (T_20_ decreases of around 20 °C from YG3S_f_ to YG15S_f_). These different behaviors confirm the existence of complex interactions between sepiolite, glycerol and starch chains. Finally, the residual weights are consistent with the inorganic fraction of the samples, increasing with the filler amount.

## 4. Conclusions

In this study, the effect of starch processing (i.e., starch gelatinization, addition of glycerol as plasticizer, casting to obtain films) and the impact of different filler amounts on the starch microstructure were evaluated through SS-NMR, XRD and FTIR-ATR techniques. While the crystallinity of the pristine starch was about 45% during processing, after gelatinization, starch became amorphous and remained amorphous also after the addition of glycerol and fillers. The obtained bio-nanocomposite films were transparent in the visible range, and the filler was uniformly distributed in the starch matrix. In the bio-nanocomposites, a strong interaction between plasticizers and starch hydroxyl groups was highlighted by FTIR-ATR analysis. The polysaccharide interacted also with sepiolite, inducing a preferential orientation of the filler along crystallographic planes {0k0}, as suggested by XRD analyses. Moreover, glycerol interacted with sepiolite by partially substituting zeolitic water present inside sepiolite’s channel. While plasticization with glycerol decreased the thermal stability of starch, the addition of sepiolite allowed recovering of the initial thermal resistance. Furthermore, the transparency of the composite films in the visible range was not affected by the highest amount of sepiolite filler. The present study demonstrates the existence of complex interactions between sepiolite, glycerol and starch chains that must be totally understood to tune the final properties of the composite materials.

## Figures and Tables

**Table 1:** Samples list and labelling (Y = yuca starch, G = glycerol, S = sepiolite). Percentages are with respect to starch content.

Sample	Glycerol (% *w*/*w*)	Sepiolite (% *w*/*w*)	Sample Form
Y_p_	0	0	Powder
Y_f_	0	0	Film
YG_f_	40	0	Film
YG3S_f_	40	3	Film
YG5S_f_	40	5	Film
YG10S_f_	40	10	Film
YG15S_f_	40	15	Film
Y5S_f_	0	5	Film
Y10S_f_	0	10	Film

**Table 2:** Thermogravimetric analysis results: decomposition temperatures T_5_ and T_20_ (i.e., the temperature at which the sample weight losses are 5% and 20%, respectively) and the residual weights at 1000 °C.

Sample	T_5_ (°C)	T_20_ (°C)	Residue (%)
Y_f_	102	286	10.94
YG_f_	156	257	5.90
YG3S_f_	197	286	10.83
YG5S_f_	200	286	12.68
YG10S_f_	197	277	17.39
YG15S_f_	194	267	20.40

## Data Availability

The data presented in this study are available on request from the corresponding authors.

## Supplementary Materials

The following supporting information can be downloaded at: https://www.mdpi.com/article/10.3390/polym15051207/s1, Figure S1: SEM images of sepiolite clay filler; Figure S2: profile fitting of C1 region in Y_p_ ^13^C CPMAS NMR spectrum; Figure S3: profile fitting of XRD diffractogram of Y_p_; Table S1: main FTIR-ATR peak assignment of film samples; Figure S4: profile fitting of FTIR 1200–800 cm^−1^ region in Y_p_; Figure S5: results of FTIR 1040/1016 peak area ratio for all the samples analyzed; Figure S6: ^29^Si CPMAS NMR spectra of neat sepiolite in comparison with Y5S_f_ and Y15S_f_ without glycerol; Figure S7: FTIR-ATR of sepiolite filler. References [41,42] are from supplementary materials.
