# Supplementary material for: Structure of Starch–Sepiolite Bio-Nanocomposites: Effect of Processing and Matrix–Filler Interactions
Source: Polymers (Basel). 2023 Feb 27;15(5):1207. doi: 10.3390/polym15051207 (PMC10007023; doi:10.3390/polym15051207)
Supplement: Supplementary file 1 [file polymers-15-01207-s001.zip › polymers-2223511-supplementary.pdf]

Supplementary Material

Structure of Starch-Sepiolite Bio-Nanocomposites: Effect of Processing and Matrix-Filler Interactions

Daniele Bugnotti [1], Sara Dalle Vacche [2,3], Leandro Hernan Esposito [2], Emanuela Callone [1], Sara Fernanda Orsini [4], Riccardo Ceccato [1], Massimiliano D'Arienzo [4], Roberta Bongiovanni [2,3], Sandra Dirè [1,*] and Alessandra Vitale [2,3,*]

1 University of Trento, Department of Industrial Engineering, 38123 Trento, Italy

2 Politecnico di Torino, Department of Applied Science and Technology, 10129 Torino, Italy

3 INSTM – Politecnico di Torino Research Unit, 50121 Firenze, Italy

4 University of Milano-Bicocca, Department of Materials Science, 20125 Milano, Italy

* Correspondence: sandra.dire@unitn.it (SD); alessandra.vitale@polito.it (AV)

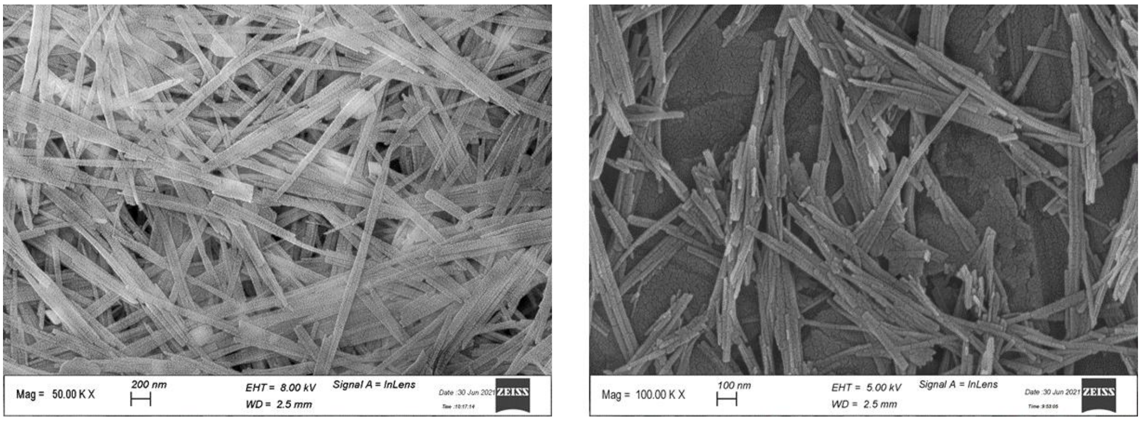

Figure S1. SEM images of sepiolite clay filler.

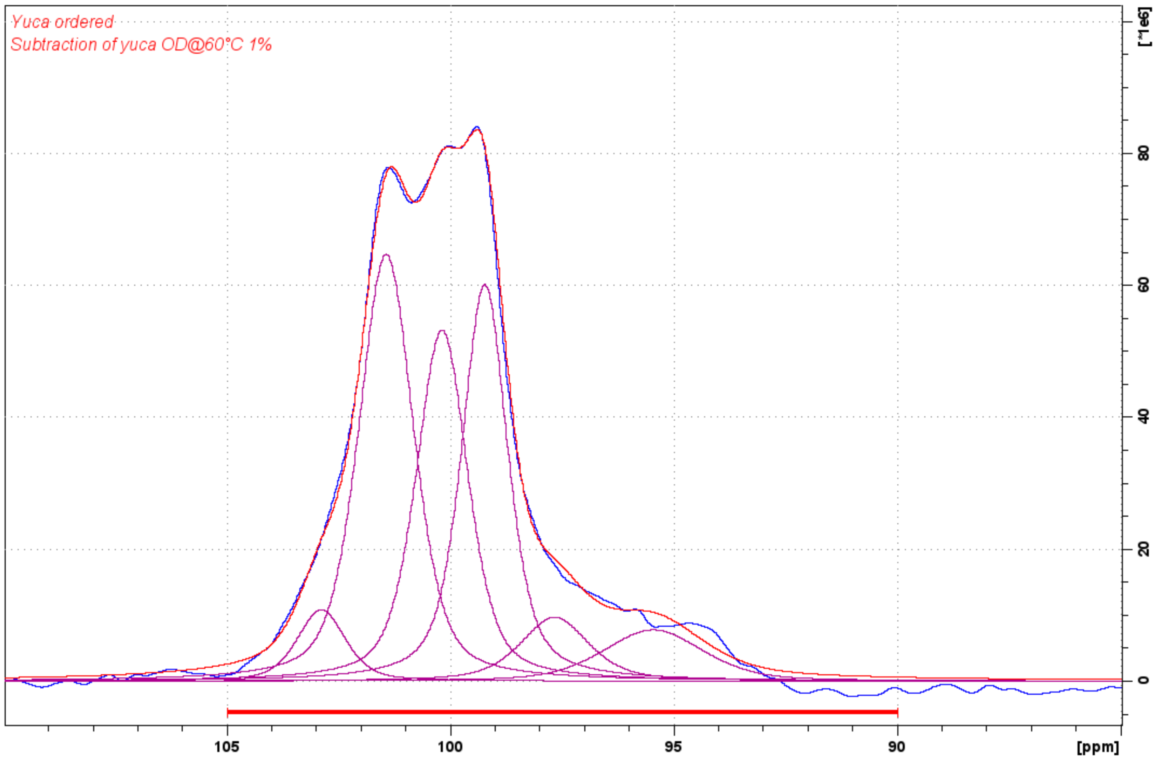

Figure S2. Profile fitting of C1 region in Y_p ^{13}C CPMAS NMR spectrum. Components at 97.7 and 95.5 ppm were attributed to interfacial conformations [1].

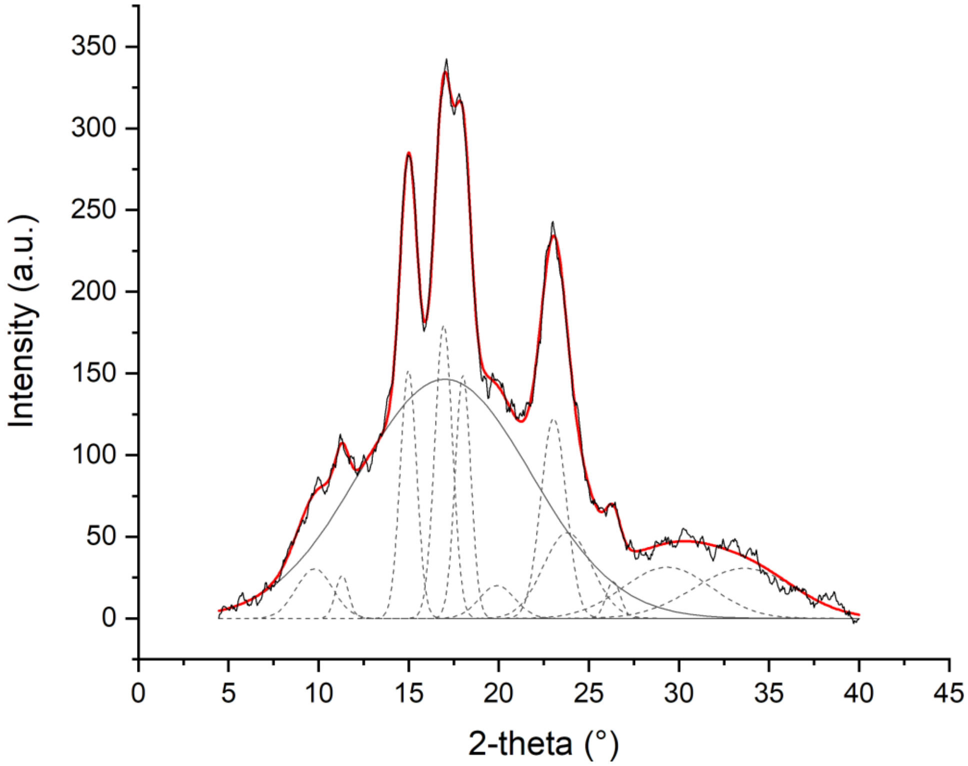

Figure S3. Profile fitting of XRD diffractogram of Y_p. The dashed peaks represent crystalline reflections while the solid line accounts for the amorphous halo.

Table S1. Main FTIR-ATR peak assignment of film samples. ν=stretching; δ=bending.

Peak	Assignment	References
3290-3280	νOH	[2]
2925	νCH_2	[2]
2884	νCH_2	[2]
1646	δOH (water scissoring)	[2,3]
1455	δCH_2	[3]
1335	δCOH	[3]
1240	CH_2OH	[2,3]
1150-1103	νCO,νCC in COH group	[4-6]
1078-926	δCOH and CH_2 related modes	[4-6]
860	COC, CH deformations	[4]
>800	skeletal vibrations	[7]

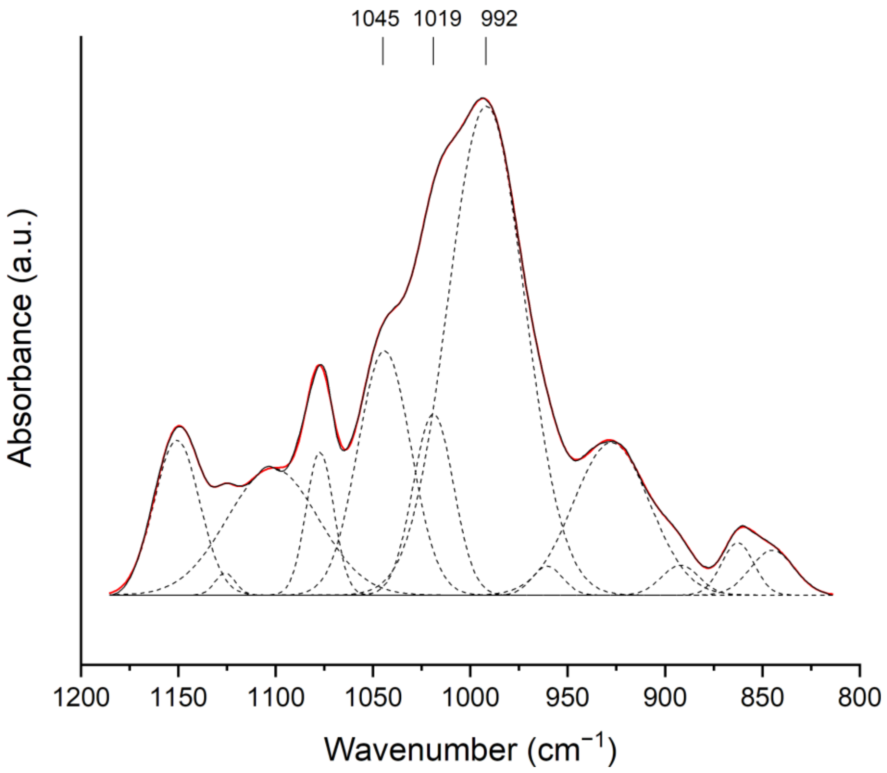

Figure S4. Profile fitting of FTIR-ATR 1200-800 cm^{-1} region in Y_p. Peak positions related to crystalline/amorphous regions are labelled.

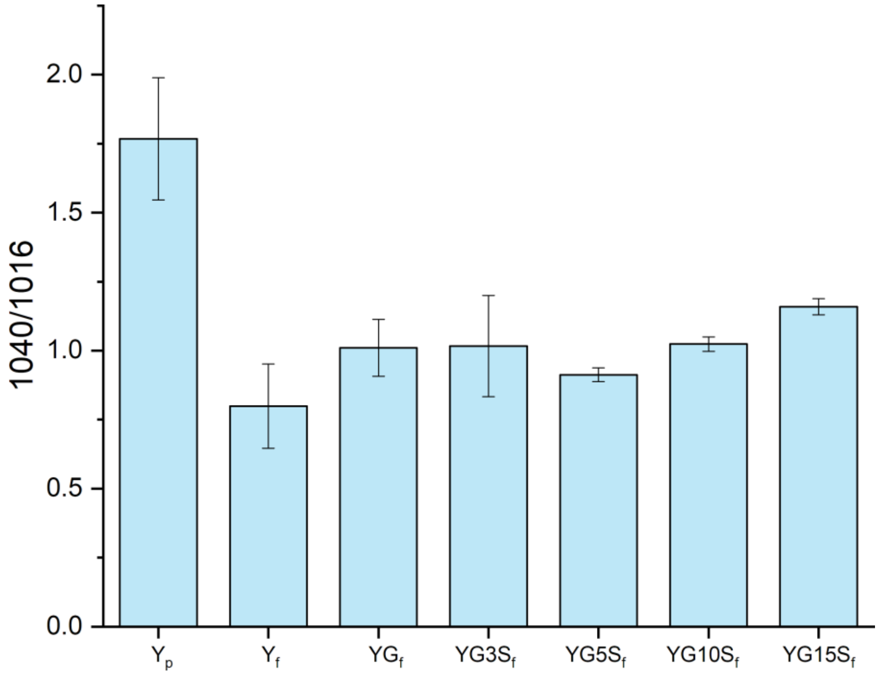

Figure S5. Results of FTIR-ATR 1040/1016 peak area ratio for all the samples analyzed.

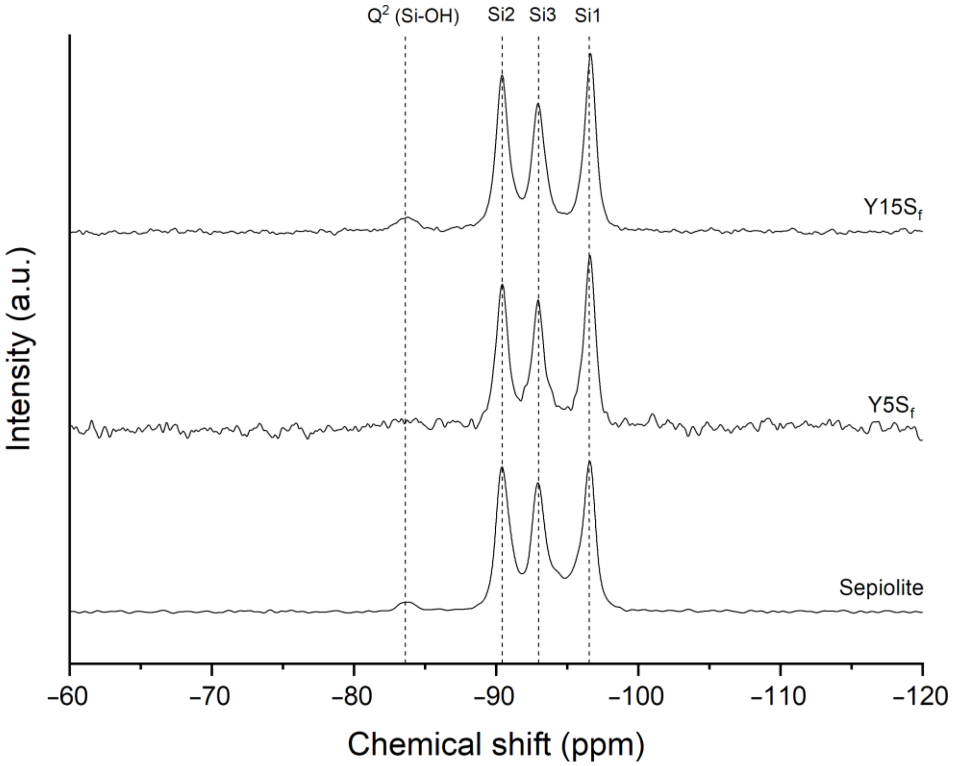

Figure S6. ^{29}Si CPMAS NMR spectra of neat sepiolite in comparison with $Y5S_f$ and $Y15S_f$ without glycerol. Intensity peak ratio was similar for the three samples.

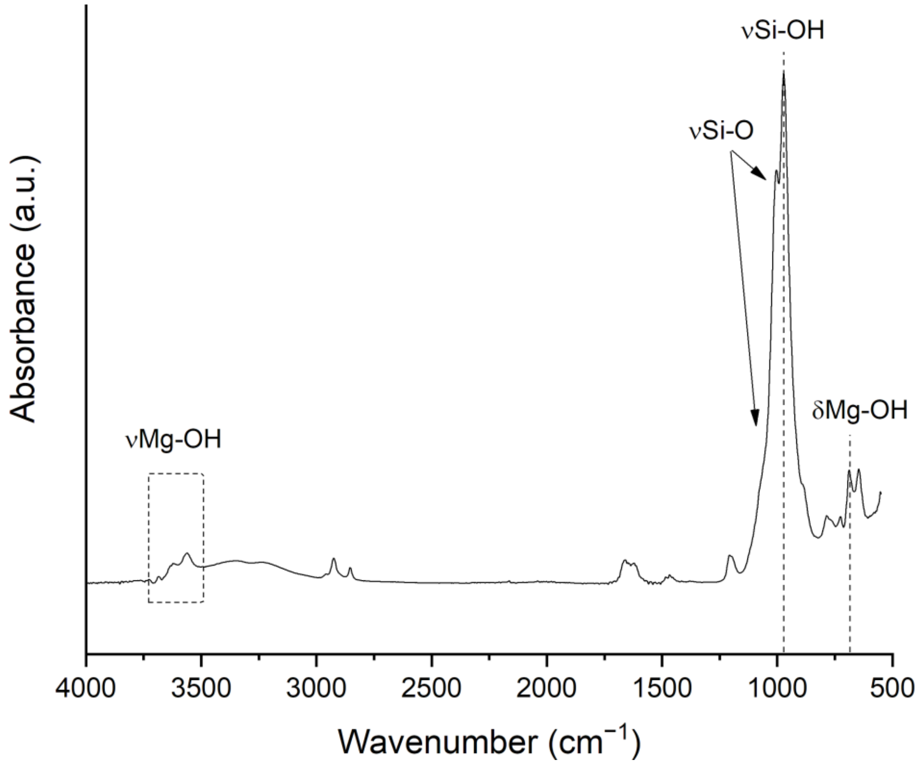

Figure S7. FTIR-ATR of sepiolite filler.

References

1. Mutungi, C.; Passauer, L.; Onyango, C.; Jaros, D.; Rohm, H. Debranched cassava starch crystallinity determination by Raman spectroscopy: Correlation of features in Raman spectra with X-ray diffraction and 13C CP/MAS NMR spectroscopy. *Carbohydr. Polym.* **2012**, *87*, 598-606.
2. Kizil, R.; Irudayaraj, J.; Seetharaman, K. Characterization of Irradiated Starches by Using FT-Raman and FTIR spectroscopy. *ournal of Agricultural and Food Chemistry* **2002**, 3912-3918.
3. Vicentini, N.M.; Dupuy, N.; Leitzelman, M.; Cereda, M.P.; Sobral, P.J.A. Prediction of Cassava Starch Edible Film Properties by Chemometric Analysis of Infrared Spectra. *Spectroscopy Letters* **2005**, 749–767.
4. van Soest, J.J.G.; Tournois, H.; de Wit, D.; Vliegenthart, J.F.G. Short-range structure in (partially) crystalline potato starch determined with attenuated total reflectance Fourier-transform IR spectroscopy. *Carbohydr. Res.* **1995**, 201-214.
5. Warren, F.J.; Gidley, M.J.; Flanagan, B.M. Infrared spectroscopy as a tool to characterise starch ordered structure - a joint FTIR-ATR, NMR, XRD and DSC study. *Carbohydrates Polymers* **2016**, 35-42.
6. Mutungi, C.; Onyango, C.; Doert, T.; Paasch, S.; Thiele, S.; Machill, S.; Jaros, D.; Rohm, H. Long- and short-range structural changes of recrystallised cassava starch subjected to in vitro digestion. *Food Hydrocolloids* **2011**, 477-485.
7. Capron, I.; Robert, P.; Colonna, P.; Brogly, M.; Planchot, V. Starch in rubbery and glassy states by FTIR spectroscopy. *Carbohydr. Polym.* **2007**, 249–259.
